# The association of Lewy bodies with limbic-predominant age-related TDP-43 encephalopathy neuropathologic changes and their role in cognition and Alzheimer’s dementia in older persons

**DOI:** 10.1186/s40478-021-01260-0

**Published:** 2021-09-25

**Authors:** Sonal Agrawal, Lei Yu, Sukriti Nag, Konstantinos Arfanakis, Lisa L. Barnes, David A. Bennett, Julie A. Schneider

**Affiliations:** 1 Rush Alzheimer’s Disease Center, Rush University Medical Center, 1750 W. Harrison street, Suite 1000, Chicago, IL 60612 USA; 2 Department of Pathology, Rush University Medical Center, Chicago, IL USA; 3 Department of Biomedical Engineering, Illinois Institute of Technology, Chicago, IL USA; 4 Department of Neurological Sciences, Rush University Medical Center, Chicago, IL USA

**Keywords:** Limbic-predominant age-related TDP-43 encephalopathy, Lewy bodies, Aging, Cognition, Alzheimer’s dementia

## Abstract

Lewy bodies (LBs) and limbic-predominant age-related TDP-43 encephalopathy neuropathologic change (LATE-NC) are common in older persons and associated with cognitive impairment. However, little is known about the relationship between LBs and LATE-NC and their combined roles in cognitive impairment and Alzheimer’s dementia in community-dwelling participants. The study included 1670 community-based participants (mean age-at-death, 89.5 years (SD = 6.65); 69% females) who underwent annual assessments of cognition to create summary measures of global cognition and cognitive domains and evaluation for Alzheimer’s dementia. Systematic neuropathologic evaluations were performed to assess LBs, LATE-NC, and Alzheimer’s disease (AD) pathology. We excluded cases with pathologically confirmed frontotemporal lobar degeneration in this study. Logistic and linear regression analyses were used, adjusted for demographics and AD pathology. LBs were present in 428 (25.6%) decedents (29 nigra-predominant, 165 limbic-type, and 234 neocortical-type) while 865 (51.7%) decedents exhibited LATE-NC (307 stage 1, 167 stage 2, and 391 stage 3). LBs combined with LATE-NC were common (15% of all participants) and in those with Alzheimer’s dementia (25%). Neocortical-type, but not nigral-predominant or limbic-type LBs increased the odds of stage 2/3 LATE-NC (odds ratio = 1.70; 95% confidence interval = 1.26–2.30). The association between neocortical-type LBs and stage 2/3 LATE-NC was stronger in those under 90 years of age and in women. In analyses of cognition and Alzheimer’s dementia, LATE-NC and neocortical-type LBs, separately, were related to lower global cognition, five specific cognitive domains, and an increased odds of Alzheimer’s dementia, above and beyond the AD pathology. Limbic-type LBs were related to lower global cognition, and the domains of episodic, working, and semantic memory, and increased odds of Alzheimer’s dementia. Furthermore, there was no interaction between limbic/neocortical-type LBs and LATE-NC on cognitive function, cognitive domains, or Alzheimer’s dementia. These findings suggest that neocortical-type LBs are associated with LATE-NC, specifically in the younger old and in women. Limbic/neocortical-type LBs and LATE-NC have separate and additive effects on cognitive function and odds of Alzheimer’s dementia.

**Supplementary Information:**

The online version contains supplementary material available at 10.1186/s40478-021-01260-0.

## Introduction

It is well recognized that the brains of older people who live in their communities have mixed or comorbid pathologies that account for most cases of Alzheimer’s dementia [37, 39, 42]. Lewy bodies (LBs) are a well-recognized co-morbid proteinopathy, frequently co-occurring with Alzheimer’s disease (AD) pathology that adds to the likelihood of dementia in persons with Alzheimer’s dementia [26, 41]. More recently, limbic predominant age-related transactive response DNA binding protein of 43 kDa (TDP-43) encephalopathy-neuropathological change (LATE-NC) predominantly in the limbic areas with or without co-existing hippocampal sclerosis (HS) has been recognized as a common pathology in older persons [34]. Increasing evidence suggests that LATE-NC is an important contributor to cognitive decline and Alzheimer’s dementia and produces an Alzheimer’s-like clinical and neuroimaging presentation including prominent episodic memory impairment [34] and progressive hippocampal atrophy[21]. Pathologically, it commonly co-occurs with HS [31, 33] and AD pathology [20, 22]. However, it is also reported in the aging brain without other pathologies [27, 30, 31] Notably, LBs have also been reported as common comorbidity in persons with LATE-NC [3, 10, 27, 32], yet little is known about the relationship between LATE-NC and LBs from community-based clinical-pathological studies. Given that these pathologies add variability, complexity, and heterogeneity to clinical phenotypes, it is important to understand the role of pathologic co-morbidities in aging and Alzheimer’s dementia; as they may provide clues to pathogenesis.

The role of LATE-NC and LBs on the likelihood of Alzheimer’s dementia and cognitive impairment in older persons with and without a pathologic diagnosis of AD is not well understood. While the co-existence of AD with LBs [26, 41] or AD with LATE-NC [20]appears to be additive rather than synergistic in the likelihood of dementia, it is not clear whether the adverse effects of LATE-NC and LBs on the likelihood of Alzheimer’s dementia are additive or synergistic.

We used clinical and neuropathology data from persons in three longitudinal clinical–pathologic studies of aging and AD: the Religious Orders Study (ROS), the Rush Memory and Aging Project (MAP), and the Minority Aging Research Study (MARS) to explore the relationship of nigral-predominant, limbic, and neocortical-type LBs with LATE-NC independent of age-at-death, sex, education, and AD pathology. Next, we tested the hypothesis that LBs interacts with LATE-NC to lower global cognition and cognitive domains proximate-to-death. Additional analyses examined the effect of LATE-NC with LBs on the odds of Alzheimer’s dementia.

## Methods

### Participants

This study included decedents from three longitudinal clinical-pathologic studies of aging and dementia, the Religious Orders Study; ROS [8], the Rush Memory and Aging Project; MAP [8] and the Minority Aging Research Study; MARS [6]. All three studies were approved by an Institutional Review Board of Rush University Medical Center and all participants signed informed consent. Brain donors, all ROS and MAP participants, and about half of MARS participants, signed an Anatomic Gift Act. Each participant also agreed to an annual clinical evaluation. There were 1491 persons enrolled in the ROS, 2198 enrolled in MAP, and 790 enrolled in MARS who had a baseline clinical evaluation. Throughout the study, 2318 persons died, 1829 of those underwent a brain autopsy (78.90% autopsy rate). To exclude potential sources of bias, we excluded those that had a pathological diagnosis of frontal temporal lobar degeneration (FTLD), including FTLD-TDP (n = 4) and other FTLD-tauopathies (n = 7) and analyses were conducted in the remaining 1670 decedents who had complete postmortem neuropathology data for LATE-NC and Lewy bodies.

### Clinical assessment of cognitive function and Alzheimer’s Dementia

A uniform structured clinical evaluation, which included a medical history, neuropsychological testing, and a neurological examination was performed in each participant at baseline and annually during follow-up. Cognitive function was evaluated using a standardized battery of nineteen neuropsychological tests (7 tests of episodic memory, 3 tests of semantic memory, 3 tests of working memory, 4 tests of perceptual speed, and 2 tests of visuospatial ability). Composite measures of 5 separate cognitive domains and global cognition were obtained, as previously described [6, 8]. The global cognition measure was computed by standardization of the raw scores of the individual tests to z scores using the baseline mean and standard deviation and averaged together while summary scores of the five specific cognitive domains were derived by averaging the z scores from tests in a specific domain.

A board-certified neurologist, blinded to pathologic data determined the clinical diagnosis of Alzheimer’s dementia proximate-to-death based on the recommendation of the Joint Working Group of the National Institute of Neurological and Communicative Disorders and Stroke and the Alzheimer’s Disease and Related Disorders Association, as described previously [28]. These criteria are consistent with our previous work as well as with the majority of work to date on the clinical syndrome of Alzheimer's dementia [11, 20]. For this study, we used a dichotomous variable for the presence vs. absence of Alzheimer’s dementia where the term Alzheimer’s dementia refers to those with either probable or possible AD dementia. This designation was made without the use of biomarkers.

### Neuropathologic examination

The mean post-mortem interval was 9.69 h (SD = 8.96). Brain autopsies were performed using standard procedures, as previously described [31]. In brief, slabs from one cerebral hemisphere were placed in a − 80° C freezer. Slabs from the contralateral hemisphere were fixed in 4% paraformaldehyde and stored in 20% glycerol and 2% DSMO. For neuropathological assessment, we used paraffin-embedded 6 µm sections from the following regions: midfrontal, middle temporal, inferior parietal, anterior cingulate, and entorhinal cortices, amygdala, mid hippocampus, and midbrain. All cases were systematic and uniformly reviewed by one of two board-certified neuropathologists (JAS and SN; authors), and one trained neuropathology researcher over the past 25 years at the laboratory of the Rush Alzheimer’s Disease Center for accurate neuropathologic assessment, blinded to clinical data.

#### Lewy bodies

Lewy bodies (LBs) were assessed in the following brain regions including midfrontal, middle temporal, inferior parietal, entorhinal, and anterior cingulate cortices, amygdala, and substantia nigra. Immunohistochemistry with alpha-synuclein (Zymed LB 509; 1:50; pSyn, 1:20,000; Wako Chemicals) was performed to detect LBs. Each case with the presence of LBs was assigned to either nigral predominant, limbic, or neocortical types as described previously [41]. In the case with nigral predominant-type, LBs were only present in the substantia nigra without evidence of LBs in the limbic or neocortical areas while in the limbic-type LBs included cases with LBs positivity in the anterior cingulate cortex or entorhinal cortex with an absence of Lewy bodies in the neocortex. Finally, cases identified with neocortical-type LBs required the presence of LBs in the midfrontal, mid temporal, or inferior parietal cortices.

#### Limbic predominant age-related TDP-43 encephalopathy neuropathological change (LATE-NC)

TAR DNA-binding protein 43 (TDP-43), a pathology marker of LATE-NC, was assessed by immunostaining 6-µm sections using phosphorylated monoclonal TAR5P-1D3, pS409/410 antibodies (before 2015; Ascenion, Munich, Germany, dilution 1:100 and since 2015; Millipore Sigma, Burlington, MA, dilution 1:400) from eight brain regions the amygdala, hippocampus CA1/subiculum, hippocampus dentate gyrus, entorhinal, midfrontal, middle temporal, anterior temporal tip, and inferior orbital frontal cortices as described previously [31]. Each sample was reviewed for the presence of TDP-43 cytoplasmic inclusions, and severity was determined on a 6-point scale based on the number of inclusions in a 0.25 mm^2^ area of greatest density as described previously [31]. Based on the consensus criteria for a LATE disease, four stages of LATE-NC progression were recognized. In stage 0, TDP-43 was absent; in stage 1, TDP-43 inclusions were localized to the amygdala; in stage 2, there was an extension to the hippocampus and/or entorhinal cortex while in stage 3, there was an extension to any of the assessed neocortical regions, as previously described [2, 30]. For descriptive analyses [2], we used the four levels of LATE-NC stages while for analytical analyses, we dichotomized LATE-NC into none/mild (stage 0 and stage 1) and moderate/severe (stages 2–3) as our earlier study [20] reported that LATE-NC stage 1 is not related to cognitive impairment and Alzheimer’s dementia.

#### Alzheimer’s disease (AD) pathology

Five brain regions including the frontal, temporal, parietal, and entorhinal cortices and hippocampus were assessed for each AD pathology marker (neuritic plaques, diffuse plaques, and neurofibrillary tangles) using a modified Bielschowsky silver stain. All three neuropathological markers of AD were counted at the highest density area in each region by board-certified neuropathologists or trained technicians blinded to all clinical data and interrater reliability on 40 cases was high (r = 0.89 to 0.93), as previously assessed elsewhere [9]. For each marker, region-specific raw counts were first divided by the standard deviation of the raw counts of the entire deceased ROS, MAP, and MARS participants. The scaled score was then averaged across the assessed regions to develop a summary score for diffuse plaques, neuritic plaques, and neurofibrillary tangles. We then averaged the summary scores of the three AD markers to obtain the global AD summary score for each subject as described previously [40]. The global AD pathology summary measure has been extensively used in previous studies and found to be a reliable method of summarizing the traditional pathologic hallmarks of AD by comparing it with other established methods commonly used to stage and classify AD pathology, including the Consortium to Establish a Registry for Alzheimer’s Disease (CERAD) system [29], Braak staging [12], and National Institute on Aging-Reagan (NIA-Reagan) criteria [1]. Since Thal phases (Aβ immunohistochemistry) were unavailable to determine pathologic diagnosis of AD using National Institute on Aging-Alzheimer’s Association (NIA-AA) criteria, modified NIA-Reagan criteria were used to determine the pathologic diagnosis of AD where a final pathologic diagnosis of AD required either intermediate or high likelihood AD as described previously [18].

#### Hippocampal sclerosis (HS)

Hippocampal sclerosis was evaluated as significant neuronal loss and gliosis at CA1 or subiculum sectors of the mid hippocampus and scored as absent and present [31].

#### APOE genotyping

APOE genotyping was determined by sequencing rs429358 (codon 112) and rs7412 (codon 158) at exon 4 of the APOE gene as described earlier [45].

## Statistical analysis

Analysis of variance or chi-square tests were used to compare demographics, clinical, and neuropathologic characteristics of subjects across LATE-NC stages.

In a logistic regression model, we examined the association between types of LBs and LATE-NC. In this model, the presence of LATE-NC was a binary outcome where LATE-NC stage 0 and 1 represented as none-mild LATE-NC while LATE-NC stage 2 and 3 represented as moderate-to-severe LATE-NC, and the main predictor of LBs was coded as three indicator variables, representing neocortical-type, limbic-type, and nigral-predominant type LBs separately. The model was adjusted for age-at-death, sex, education, and AD pathology (core model). Next, we repeated the model by including a term for APOE ε4.

Next, we examined whether the association between LB types and LATE-NC differed by oldest old. To do so, we created a binary variable for age (age-at-death 90 years and above, with 89 years and below as the reference group) and reran the core models by including two-way interaction terms for age and LB types. A similar analysis was conducted to examine whether the association between LB types and LATE-NC differed by sex. Then we repeated the core model and stratified the cohort by age group and sex.

Separately, we conducted linear regression to examine the relation of LBs and LATE-NC pathologies to the level of global cognitive function proximate to death and separately the measures of episodic, semantic, and working memory, perceptual speed, and visuospatial skills. In brief, we first examined the relationship between LATE-NC and cognitive function and then added terms for the presence of nigral predominant, limbic and neocortical-types LBs and finally added interaction term for LBs and LATE-NC. These steps were repeated for the specific measures of five different cognitive domains.

Finally, we ran a similar set of analyses that used logistic regression analysis to examine the association of LATE-NC and LBs with the odds of Alzheimer’s dementia. In these models, Alzheimer’s dementia diagnosis at death was the binary outcome, and we first included the terms for demographics, AD, and LATE-NC to examine the relation between LATE-NC and Alzheimer’s dementia proximate to death. We then added terms for the nigral- predominant type, limbic-type, and neocortical-type LBs to examine if any type of LBs independently increased the odds of Alzheimer’s dementia. Finally, to test whether the association of LATE-NC and LBs is additive or synergistic; we repeated the model by adding an interaction term for LATE-NC and LBs.

Analyses were performed using SAS/STAT software, version 9.4 for Linux (SAS Institute Inc, Cary, NC), and statistical significance for all analyses was determined at α level of 0.05.

## Results

Demographic, clinical, and neuropathologic characteristics of participants (n = 1670) are represented in Table 1. The mean age-at-death was 89.5 years (range between 65.9 and 108.2 years), 524 (31%) were men, 1146 (69%) were females, and 96% were non-Latino white. Nearly half of the subjects (n = 714) had Alzheimer’s dementia and one-fourth (n = 412) had APOE ε4 allele.

**Table 1:** Demographics, clinical and neuropathologic characteristics of participants across LATE-NC stages

Characteristics		Total (n = 1,670)	LATE-NC				Estimate, *p*-value^a^
			Stage 0 (n = 805)	Stage 1 (n = 307)	Stage 2 (n = 167)	Stage 3 (n = 391)	
Demographics							
Age-at-death, mean (SD) yrs		89.55 (6.65)	87.86 (6.95)	89.94 (6.34)	91.39 (5.88)	91.93 (5.52)	F_3,1666_ = 41.32, *p* < 0.001
Female, n (%)		1146 (68.62)	531 (65.96)	205 (66.78)	116 (69.46)	294 (75.19)	χ2 = 11.02, df = 3, *p* = 0.011
Education, mean (SD) yrs		16.19 (3.60)	16.34 (3.80)	16.21 (3.43)	16.29 (3.62)	15.83 (3.27)	F_3,1666_ = 1.85, *p* = 0.136
*APOE ε4, n (%)		412 (25.37)	161 (20.64)	65 (21.67)	53 (32.52)	133 (34.91)	χ2 = 34.08, df = 3, *p* < 0.001
Clinical							
Global cognitive score; lv, mean (SD)		− 1.00 (1.20)	− 0.71 (1.11)	− 0.87 (1.11)	− 1.22 (1.25)	− 1.62 (1.20)	F_3,1660_ = 58.89, *p* < 0.001
Episodic memory; lv, mean (SD)		− 0.93 (1.40)	− 0.55 (1.28)	− 0.76 (1.33)	− 1.18 (1.40)	− 1.75 (1.33)	F_3,1659_ = 75.60, *p* < 0.001
Semantic memory; lv, mean (SD)		− 1.28 (1.68)	− 0.91 (1.45)	− 1.15 (1.55)	− 1.55 (1.84)	− 2.04 (1.88)	F_3,1663_ = 44.85, *p* < 0.001
Working memory; lv, mean (SD)		− 0.75 (1.13)	− 0.58 (1.08)	− 0.69 (1.07)	− 0.91 (1.13)	− 1.10 (1.19)	F_3,1660_ = 20.18, *p* < 0.001
Perceptual speed; lv, mean (SD)		− 1.11 (1.06)	− 0.95 (1.07)	− 1.08 (1.04)	− 1.20 (1.01)	− 1.43 (1.00)	F_3,1645_ = 18.94, *p* < 0.001
Visuospatial ability; lv, mean (SD)		− 0.57 (1.07)	− 0.43 (1.02)	− 0.50 (1.10)	− 0.69 (1.12)	− 0.84 (1.09)	F_3,1640_ = 13.84, *p* < 0.001
^+^ Alzheimer’s dementia, n (%)		714 (43.43)	248 (31.47)	119 (39.27)	79 (48.17)	268 (68.89)	χ2 = 152.16, df = 3, *p* < 0.001
Neuropathologic							
AD (NIA-Reagan), n (%)		1084 (64.91)	448 (55.65)	205 (66.78)	112 (67.07)	319 (81.59)	χ2 = 78.83, df = 3, *p* < 0.001
AD pathology score, mean (SD)		0.75 (0.60)	0.61 (0.54)	0.76 (0.63)	0.84 (0.68)	1.01 (0.67)	F_3,1660_ = 38.34, *p* < 0.001
LBs, n (%)	Nigral predominant type	29 (1.75)	16 (2.01)	6 (1.97)	2 (1.21)	5 (1.28)	χ2 = 23.82, df = 9, p = 0.004
	Limbic-type	165 (9.88)	72 (9.06)	33 (10.82)	14 (8.48)	46 (11.79)	
	Neocortical-type	234 (14.14)	86 (10.82)	42 (13.77)	34 (20.61)	72 (18.46)	
^^^HS, n (%)		162 (9.74)	13 (1.62)	10 (3.28)	16 (9.58)	123 (31.54)	χ2 = 285.53, df = 3, *p* < 0.001

^a^Estimates are either F_degrees of freedom, observations _ values derived from ANOVA tests or chi-square (degrees of freedom)

^*^Data missing for 46 participants; ^+^data missing for 26 participants; ^^^data missing for 6 participants

Abbreviations: AD-Alzheimer’s disease;; lv-last evaluation; HS-hippocampal sclerosis; LATE-NC-limbic-predominant age-related TDP-43 encephalopathy neuropathologic change; LBs-Lewy bodies; yrs-years

LB pathology was present in 428 (25.6%) of decedents, of which 29 (1.7%) had nigral predominant-type LBs, 165 (9.8%) had limbic-type LBs, and 234 (14.0%) had neocortical-type LBs. The frequency of APOE ε4 allele was significantly higher in people with LBs than those without LBs (29.43% vs 23.89%, χ2 = 5.16, df = 1, *p* = 0.023). AD pathology was common; the frequency of pathologic diagnosis of Alzheimer’s disease was more frequent in people with LBs compared to those without LBs (71.56 vs 62.51%, χ2 = 11.69, df = 1, *p* = 0.006).

More than half of the decedents (51.7%) had TDP-43 inclusions in any of the assessed brain regions, of which 307 (18.3%) had stage 1, 167 (10%) had stage 2, and 391 (23.4%) had stage 3. The frequency of APOE ε4 allele was significantly higher in those with LATE-NC than those without LATE-NC (30.3% vs 20.6%). AD pathology commonly coexisted in those with LATE-NC. Approximately 56% of individuals without LATE-NC had a pathologic diagnosis of Alzheimer’s disease, and the percentage was higher for those with LATE-NC (73.52%). Hippocampal sclerosis was common in those with LATE-NC (17.22%) and was uncommon in those without LATE-NC (1.62%). Another pathology commonly coexisting in those with LATE-NC was LBs. From 1670 subjects, two hundred fifty-four (30%) participants with LATE-NC and 21% of participants without LATE-NC had LBs in any region. The regional distribution of LBs was similar in those with and without LATE-NC, except there was a higher proportion of neocortical-type LBs in those with LATE-NC (17.1%) than those without LATE-NC (10.6%).

### Association of Lewy bodies with LATE-NC pathology

To determine the association of LB types with LATE-NC, a logistic regression analysis was performed controlling for age-at-death, sex, education, and AD pathology. Compared to individuals without LBs, neocortical-type LBs were associated with increased odds of LATE-NC (OR = 1.70; 95% CI = 1.26–2.30; *p* < 0.001). We did not find an association of nigral predominant type (*p* = 0.587) or limbic-type (*p* = 0.920) LBs with the odds of LATE-NC (Table 2, model 1). In the secondary analysis that further controlled for APOE ε4, the association between neocortical-type LBs and LATE-NC persisted (Table 2, model 2). In sensitivity analyses, we ran similar logistic regression models, adjusted for neuritic plaques and neurofibrillary tangles burden separately. We find a significant association remained between neocortical-type LBs and LATE-NC (Additional file 1: Table S1).

**Table 2:** Relation of Lewy bodies with LATE-NC

Predictor	Outcome: LATE-NC Odds ratio (95% confidence intervals), p-value	
	Model 1	Model 2
Age-at-death	1.08 (1.06, 1.10), *p* < 0.001	1.09 (1.07, 1.11), *p* < 0.001
Male sex	0.89 (0.70, 1.14), *p* = 0.382	0.88 (0.68, 1.13), *p* = 0.334
Education	0.99 (0.96, 1.02), *p* = 0.755	0.98 (0.95, 1.02), *p* = 0.495
Nigral predominant-type LBs	0.78 (0.31, 1.91), *p* = 0.587	0.79 (0.32, 1.96), *p* = 0.621
Limbic-type LBs	1.01 (0.71, 1.45), *p* = 0.920	1.01 (0.70, 1.45), *p* = 0.953
Neocortical-type LBs	1.70 (1.26, 2.30), *p* < 0.001	1.72 (1.26, 2.34), *p* < 0.001
AD pathology	2.09 (1.76, 2.49), *p* < 0.001	1.85 (1.53, 2.22), *p* < 0.001
APOE ε4		1.83 (1.40, 2.37), *p* < 0.001

Model 1 was adjusted for age-at-death, sex, education, and AD pathology

Model 2 was adjusted for age-at-death, sex, education, AD pathology, and APOE ε4

Next, we included interaction terms for age and sex separately to examine whether the relationship between neocortical-type LBs and LATE-NC differed by demographic sub-groups that have been shown to influence the accumulation of pathology [5, 15, 16]. First, we found that the association between neocortical-type LBs and LATE-NC was stronger in participants less than 90 years of age compared to those aged 90 years and above (OR = 2.52; 95% CI = 1.63, 3.91; *p* = 0.015). Second, women showed a stronger association between neocortical-type LBs with LATE-NC compared to men (OR = 2.16; 95% CI = 1.49, 3.12; *p* = 0.029).

In stratified analyses, the association of neocortical-type LBs and LATE-NC was only present in those less than 90 years of age (age < 90 years: OR = 2.26; 95% CI = 1.43, 3.55, p < 0.001; age ≥ 90 years: OR = 1.34; 95% CI = 0.90, 2.00, i = 0.147) and women (females: OR = 2.20; 95% CI = 1.52, 3.19, *p* < 0.001; males: odds ratio = 0.99; 95% CI = 0.57–1.71, *p* = 0.977) (Additional file 1: Table S2 and S3).

Because LATE-NC and LBs pathology commonly co-existed with AD pathology reported by others and us [20, 22, 41], we performed two additional analyses to examine whether the association between neocortical-type LBs and LATE-NC remained in people with and without pathologic diagnosis of AD. First, where a term for an interaction between different types of LBs and AD pathology was added to logistic regression model with LATE-NC as outcomes. In these models, the interaction term for neocortical-type LBs and AD pathology did not reach statistical significance (*p* = 0.7015), whereas neocortical-type LBs remained associated with LATE-NC (*p* = 0.024; data not shown). Second, we ran a stratified logistic analysis in people with and without AD pathology to examine the association between neocortical-type LBs and LATE-NC, adjusted age, sex, education, and APOE ε4. The association result remained significant in both groups (people with AD: OR = 1.62; 95% CI = 1.15, 2.29, *p* = 0.0059; people without AD: OR = 2.12; 95% CI = 1.13, 3.98, *p* = 0.019) (Additional file 1: Table S4).

### Lewy bodies, LATE-NC, cognitive function, and cognitive domains

We ran a series of linear regression models, controlling for age, sex, education, and AD pathology to investigate possible separate and synergistic roles of LBs and LATE-NC on cognition. The first model examined the relationship between LATE-NC and global cognition. LATE-NC was associated with a 0.43 standard unit lower global cognitive score (Table 3, model 1). In a separate model, we then examined the relationship between LB types and global cognition. Neocortical-type LBs and limbic-type LBs, but not nigral-type Lewy bodies were associated with a 0.60 and 0.31 standard unit lower global cognitive score (Table 3, model 2). With both LATE-NC and LB types included in the same model, the effects of LATE-NC, limbic-type, and neocortical-type LBs on cognitive function were unchanged (Table 3, model 3). Finally, when terms for the interaction between LATE-NC and neocortical-type LBs and for LATE-NC and limbic-type LBs were added to the model, neither were significant (*p*’s > 0.492; data not shown), indicating that the effects of both pathologies on global cognition are additive.

**Table 3:** Relation of Lewy bodies and LATE-NC pathology to cognitive outcomes

Models	Predictor	Outcome					
		Global cognition Estimate (SE), *p*-value	Episodic memory Estimate (SE), *p*-value	Semantic memory Estimate (SE), *p*-value	Working memory Estimate (SE), *p*-value	Perceptual speed Estimate (SE), *p*-value	Visuospatial ability Estimate (SE), *p*-value
Model 1	LATE-NC	− 0.43 (0.05), *p* < 0.001	− 0.61 (0.06), *p* < 0.001	− 0.51 (0.08), *p* < 0.001	− 0.20 (0.05), *p* < 0.001	− 0.13 (0.05), *p* = 0.014	− 0.16 (0.05), *p* = 0.003
Model 2	Nigral predominant-type LBs	− 0.13 (0.19), *p* = 0.491	− 0.24 (0.22), *p* = 0.281	− 0.30 (0.27), *p* = 0.275	0.13 (0.19), *p* = 0.499	− 0.04 (0.18), *p* = 0.826	− 0.12 (0.19), *p* = 0.514
	Limbic-type LBs	− 0.31 (0.08), *p* < 0.001	− 0.26 (0.99), *p* = 0.007	− 0.55 (0.12), *p* < 0.001	− 0.21 (0.08), * p* = 0.013	− 0.15 (0.08), *p* = 0.060	− 0.11 (0.08), *p* = 0.169
	Neocortical-type LBs	-0.60 (0.07), *p* < 0.001	− 0.56 (0.08), *p* < 0.001	− 0.79 (0.10), *p* < 0.001	− 0.49 (0.07), * p* < 0.001	− 0.39 (0.07), *p* < 0.001	− 0.28 (0.07), *p* < 0.001
Model 3	LATE-NC	− 0.40(0.05), *p* < 0.001	− 0.58 (0.06), *p* < 0.001	− 0.48 (0.08), *p* < 0.001	− 0.18 (0.05), * p* = 0.001	− 0.10 (0.05), *p* = 0.052	− 0.15 (0.05), *p* = 0.007
	Nigral predominant-type LBs	− 0.15 (0.18), *p* = 0.421	− 0.27 (0.21), *p* = 0.215	− 0.32 (0.27), *p* = 0.233	0.12 (0.19), * p* = 0.527	-0.04 (0.18), *p* = 0.803	− 0.13 (0.19), *p* = 0.488
	Limbic-type LBs	− 0.31 (0.08), *p* < 0.001	− 0.26 (0.09), *p* = 0.005	− 0.55 (0.12), *p* < 0.001	− 0.21 (0.08), * p* = 0.013	− 0.15 (0.08), *p* = 0.059	− 0.11 (0.08), *p* = 0.166
	Neocortical-type LBs	− 0.56 (0.07), *p* < 0.001	− 0.49 (0.08), *p* < 0.001	− 0.74 (0.10), * p* < 0.001	− 0.47 (0.07), * p* < 0.001	− 0.38 (0.07), *p* < 0.001	− 0.26 (0.07), *p* < 0.001

Model 1 has LATE-NC as the independent variable and global cognition or 5 cognitive domains as the outcome. Model 2 has 3 types of LBs as the independent variables and global cognition or 5cognitive domains as the outcome. Model 3 has LATE-NC and LB-types as independent variables and global cognition or 5 cognitive domains as the outcome. All models were adjusted for age-at-death, sex, education, and AD pathology

There are relatively little data available on the associations of LBs or LATE-NC pathologies with cognitive domains. In separate models, LATE-NC and neocortical-type LBs were each independently associated with lower function in each of the five cognitive domains proximate to death including episodic memory, semantic memory, working memory, perceptual speed, and visuospatial ability (Table 3, model 1 and 2). Moreover, limbic-type LBs were also associated with lower episodic memory, semantic memory, and working memory but not with lower perceptual speed and visuospatial skills (Table 3, model 2). In the models with both LATE-NC and LBs, the association of LATE-NC, neocortical-type, and limbic-type LBs with cognitive domains was essentially unchanged (Table 3, model 3). Finally, we tested whether limbic or neocortical-type LBs interact with LATE-NC for the 5 cognitive domains by repeating the model with two terms, one for the interaction between LATE-NC and neocortical-type LBs, and another for LATE-NC and limbic-type LBs, adjusted for age, sex, education, and global AD pathology. The interaction between LATE-NC and neocortical-type LBs for perceptual speed was significant but not in the expected direction (estimates = 0.28, SD = 0.14, *p* = 0.042). No interactions were observed between LATE-NC and limbic/neocortical-type LBs for other cognitive domains (data not shown).

### Lewy bodies, LATE-NC, and Alzheimer’s dementia

We conducted similar sets of analyses for Alzheimer’s dementia using logistic regression models, controlling for age, sex, education, and AD pathology. The first model examined the relationship between LATE-NC and Alzheimer’s dementia. LATE-NC increased the odds of Alzheimer’s dementia by 2.22-fold (Table 4, model 1). We then examined the relationship between LB types and Alzheimer’s dementia. Neocortical-type LBs increased the odds of Alzheimer’s dementia by 3.24-fold (Table 4, model 2). Notably, limbic-type LBs were also associated with increased the odds of Alzheimer’s dementia by 1.89-fold (Table 4, model 2). There was no association between nigral predominant-type LBs and Alzheimer’s dementia. We then fit the model with LB types and LATE-NC; the effect of LATE-NC, limbic-type LBs, and neocortical-type LBs on the odds of Alzheimer’s dementia was essentially unchanged (Table 4, model 3).

**Table 4:** Relation of Lewy bodies and LATE-NC to Alzheimer’s dementia outcomes

Models	Predictors	Outcome: Alzheimer’s dementia Odds ratio (95% CI), *p*-value
Model 1	LATE-NC	2.22 (1.78, 2.85), *p* < 0.001
Model 2	Nigral predominant-type LBs	1.64 (0.72, 3.72), *p* = 0.232
	Limbic-type LBs	1.89 (1.30, 2.74), *p* < 0.001
	Neocortical-type LBs	3.24 (2.33, 4.51), *p* < 0.001
Model 3	LATE-NC	2.128 (1.71, 2.77), *p* < 0.001
	Nigral predominant-type LBs	1.74 (0.75, 4.01), *p* = 0.189
	Limbic-type LBs	1.91 (1.31, 2.79), *p* < 0.001
	Neocortical-type LBs	3.07 (2.20, 4.29), *p* < 0.001

Model 1 has LATE-NC as the independent variable and Alzheimer’s dementia as the outcome. Model 2 has types of LBs as the independent variables and Alzheimer’s dementia as the outcome. Model 3 has LATE-NC and LB-types as independent variables and Alzheimer’s dementia as the outcome. All models were adjusted for age-at-death, sex, education, and AD pathology

To test whether limbic-type or neocortical-type LBs modified the effect of LATE-NC on Alzheimer’s dementia, we repeated the model with adding two terms, one is the interaction between LATE-NC and limbic-type LBs and another is the interaction between LATE-NC and neocortical-type LBs. The interaction was not significant between limbic-type LBs and LATE-NC (*p* = 0.995; data not shown) as well as neocortical-type LBs and LATE-NC (*p* = 0.974; data not shown), suggesting that the effects of LATE-NC and limbic/neocortical-type LBs on the odds of Alzheimer’s dementia are in essence additive.

## Discussion

In this clinical-pathological study of 1670 older participants, we found that neocortical LBs and LATE-NC are common and related proteinopathies in aging and Alzheimer’s dementia. Neocortical-type LBs, but not nigral predominant or limbic-type LBs are associated with increased odds of stage 2/3 LATE-NC stages above and beyond demographics, AD pathology, and APOE ε4 allele. Interestingly, persons with age below 90 years and women are more likely to have the relationship between neocortical-type LBs and LATE-NC compared to those with advanced age over 90 years and men. The advanced stages of LATE-NC (stage 2 and above) pathology and neocortical-type LBs were each independently related to lower global cognitive function and specific 5 cognitive domains, and increased odds of Alzheimer’s dementia, above and beyond the AD pathology. Limbic-type LBs were also related to lower global cognition and specific cognitive domains (episodic memory, working memory, and semantic memory) whereas nigral-predominant LBs were not related to any specific cognitive domains. Finally, neocortical-type/limbic-type LBs and LATE-NC had separate and additive contributions to global cognition and Alzheimer’s dementia, but there was no evidence of a multiplicative effect to increase the likelihood of Alzheimer’s dementia and lower cognition beyond their additive effects.

Though LATE-NC and LBs are common in the aging brain and in those with Alzheimer’s dementia [20, 34, 41], relatively few studies have explored the co-occurrence and relationship of LBs with LATE-NC/TDP-43 proteinopathy. A study with 221 Alzheimer’s dementia cases has shown that LBs were more common in individuals with TDP-43 and about 30% of cases had both LBs and TDP-43 pathologies [7]. Another study using the Uniform Data Set from participants in the National Alzheimer’s Coordinating Center database has reported that nearly half of total participants with and without Alzheimer’s dementia had LBs and LATE-NC pathology and provided evidence that LBs, particularly limbic-type is associated with LATE-NC, adjusted for age, sex, and education [10]. This study was remarkable for the use of a large sample with extensive clinical and neuropathological data. However, analyses were not adjusted for AD pathology, a significant potential confounder, nor APOE ε4 allele. An important issue in unraveling the relationship between LBs and LATE-NC is the common coexistence of AD pathology and other potential confounders like APOE ε4 allele. Our previous work has shown TDP-43 pathology and separately LBs are common mixed neurodegenerative pathologies often in the context of a pathologic diagnosis of AD, however, the specific relationship between LATE-NC and LBs and their separate and combined effects on cognition and Alzheimer’s dementia was not studied [20]. The current study extends previous findings by examining the frequency and impact of both pathologies in the older brain after accounting for potential confounders and provides important data on a large community-based cohort. First, our study showed that nearly 30% of subjects had mixed LATE-NC and LB pathology. Second, we found that neocortical-type LBs were associated with increased odds of LATE-NC, after accounting for demographics, and AD pathology. However, only neocortical, but not nigral-predominant or limbic-type LBs were associated with LATE-NC. These findings were unchanged after controlling for APOE ε4. A possible explanation for the discrepancy between the previous study [10] and our study could be due to methodological differences in the documentation of LATE-NC group. In the present study, analyses included those with LATE-NC stage 2 and stage 3 and compared with LATE-NC stage 0 and stage 1 (the reference group) while LATE-NC stages 1–3 were compared with stage 0 in the Besser et al. study. However, a robust relationship between neocortical-type LBs and LATE-NC after accounting for potential confounders such as AD pathology suggests that neocortical-type LBs are important and more research should focus on the pathways linked with LBs to understand the underlying LATE-NC pathogenesis.

Little is known about the role of co-existing LATE-NC and LBs in cognitive function and Alzheimer’s dementia. One study derived data from 119 human postmortem brains of those with and without dementia to examine the rate of cognitive decline and mini-mental state examination score in age-matched LB cases with and without TDP-43 pathology and reported that neither group was significantly different [27]. The sample size was small and not adjusted for potential confounders, particularly AD pathology. By contrast, our results indicate that LBs and LATE-NC additively lower global cognitive function and all 5 cognitive domains (episodic memory, semantic memory, working memory, visuospatial ability, and perceptual speed) and further increase the odds of Alzheimer’s dementia. While the additive effects of both pathologies were robust, our data did not indicate that LATE-NC and LBs interact synergistically to increase the odds of Alzheimer’s dementia or lower global cognition and the cognitive domains (a trend toward a protective effect on perceptual speed is potentially spurious but follow-up studies are needed for confirmation). Further study is warranted, particularly to examine the impact of co-pathologies (LBs, LATE-NC, and AD) on the trajectory of cognitive decline to understand clinical manifestations of these pathologies in isolated forms of the disease as well as in mixed forms of the disease as described by others [23, 24].

This study indicates an additive effect of both pathologies and strongly suggests that the presence of multiple degenerative lesions deteriorates cognitive function and the likelihood of dementia. This finding is consistent with a recent study by Robinson, et al. [38]and supports the idea that targeting only AD pathologies may leave other drivers of dementia behind, such as TDP-43 or α-synuclein. Thus, a treatment targeting Alzheimer’s disease may need not only to reduce amyloid-β and tau but also may need to lower the burden of other degenerative pathologies that contributes to dementia, i.e. LATE-NC and LBs. By contrast, Robinson, et al. [38] showed mixed results when investigating the relationship between neocortical/limbic-type LBs and dementia. In the NACC cohort, neocortical/limbic-type LBs associated with dementia consistent with our study, but not in the Center for Neurodegenerative Disease Research (CNDR) cohort. The null findings in the latter cohort may be attributable to the lower mean age-at-death compared to NACC and our cohort.

It is interesting to note that the relationship between LBs and LATE-NC associations was mainly restricted to the neocortical regions. This finding demonstrates the possibility that abnormally aggregated proteins associated with Lewy body disease or LATE disease may locally interact across cortical regions. This explanation is potentially supported by a study that reported TDP-43 neuronal cytoplasmic inclusions were co-localized with neocortical-type LBs in Dementia with Lewy body disease [17]. Future studies mainly focusing on interactions between TDP-43 and alpha-synuclein at the molecular level using in-vitro approach or double or multiplex immunohistochemistry methods may be helpful to understand the pathophysiologic pathways linking both pathologies in aging. Indeed, a relationship between LBs and LATE may suggest common shared etiopathogenesis and risk factors.

It is important to understand the risk factors that may drive the relationship between two age-related non-AD neurodegenerative pathologies. There is evidence that non-AD neurodegenerative pathologies occur more frequently in men and those above 90 years [5, 15, 16, 34, 43]. However, in the current study, we found that the relationship between neocortical-type LBs and LATE-NC was mainly driven by women and young older people (< 90 years). Given that LBs are more common in men and increase with age but do not continue to increase in the oldest age [5, 15], LATE-NC is more common in women and increases with age [36], and females are normally more likely to survive to advanced age than males [5]; complicates interpretation of these findings. Therefore, further studies of coexisting LBs and LATE-NC pathology with a specific focus on age and sex are warranted. Another potential effort should be carried out in the field of genetic factors. For instance, the gene variants in GRN and TMEM106B, which are associated with increased risk of LATE-NC [14, 35, 44], are also associated with DLB [25] and can be a potential candidate for explaining the LB and LATE-NC connection.

Strengths of this study include the use of detailed, systematic, and uniform neuropathology data for both LATE-NC and Lewy bodies from three well-established large community-based cohort studies collected blinded to clinical data. This study systematically defined the LB types based on their distribution in the brain and depicts the relationship between different LB types with LATE-NC. Also, this study prospectively followed older persons with no known dementia, conducted follow-up clinical evaluations blinded to previous evaluations that resulted in detailed diagnostic data on dementia, and neuropsychological tests proximate to death. Finally, this study has high autopsy rates that minimize the risk of selection bias.

This study also has limitations. First, the study sample included mainly non-Latino whites with high levels of education; thus, this data may not generalize to more diverse populations. Further research in underrepresented groups (e.g., African Americans, Latinos, and others) is warranted to understand the relationship between LATE-NC and LB types in diverse populations. Second, this study did not include cases with amygdala-predominant type LBs recommended by new Attems/McKeith criteria [4] as well as did not characterize the LBs based on Braak LBD stages [13]. Third, we determined the pathologic diagnosis of AD pathology using NIA-Reagan criteria [18] rather than using NIA-AA criteria [19]. Finally, this study did not describe potential functional mechanisms that can explain the association of LBs with LATE-NC.

## Supplementary Information

**Additional file 1.** Supplementary table 1 for the association of neocortical-type LBs with LATE-NC, adjusted for demographics, APOE ε4, neuritic plaques, and neurofibrillary tangles burden. Supplementary tables 2–4 for the association of neocortical-type LBs with LATE-NC, stratified by age, sex, and pathologic diagnosis of AD. AD = Alzheimer’s disease; LATE-NC = limbic-predominant age-related TDP-43 encephalopathy neuropathological change; LBs = Lewy bodies.

## Data Availability

Raw data are available by request through the Rush Alzheimer's Disease Center (RADC) Research Resource Sharing Hub https://www.radc.rush.edu/.

## Abbreviations

AD: Alzheimer’s disease
APOE: Apolipoprotein E
CI: Confidence intervals
FTLD: Frontotemporal lobar degeneration
HS: Hippocampal sclerosis
LATE-NC: Limbic-predominant age-related TDP-43 encephalopathy neuropathologic change
LBs: Lewy bodies
lv: Last evaluation
MAP: Memory and Aging Project
MARS: Minority Aging Research Study
OR: Odds ratio
ROS: Religious Orders Study
TDP-43: Transactive response DNA binding protein of 43 kDa
SD: Standard deviation
