# Supplementary material for: The association of Lewy bodies with limbic-predominant age-related TDP-43 encephalopathy neuropathologic changes and their role in cognition and Alzheimer’s dementia in older persons
Source: Acta Neuropathol Commun. 2021 Sep 25;9:156. doi: 10.1186/s40478-021-01260-0 (PMC8466680; doi:10.1186/s40478-021-01260-0)
Supplement: Supplementary file 1 — Additional file 1. Supplementary table 1 for the association of neocortical-type LBs with LATE-NC, adjusted for demographics, APOE ε4, neuritic plaques, and neurofibrillary tangles burden. Supplementary tables 2–4 for the association of neocortical-type LBs with LATE-NC, stratified by age, sex, and pathologic diagnosis of AD. AD = Alzheimer’s disease; LATE-NC = limbic-predominant age-related TDP-43 encephalopathy neuropathological change; LBs = Lewy bodies. [file 40478_2021_1260_MOESM1_ESM.docx]

**Supplementary tables**

**Table 1:** **Association of Lewy bodies with LATE-NC, adjusted for demographics, neuritic plaques, neurofibrillary tangles, and** **APOE ε4 .**

| Predictor | Outcome: LATE-NC  OR (95% CI), p-value | |
| --- | --- | --- |
|  | Model 1 | Model 2 |
| Age-at-death | 1.09 (1.07, 1.11), p<0.001 | 1.09 (1.06, 1.11), p<0.001 |
| Male sex | 0.89 (0.69, 1.15), p=0.39 | 0.89 (0.69, 1.14), p=0.375 |
| Education | 0.988 (0.95, 1.02), p=0.469 | 0.99 (0.96, 1.02), p=0.635 |
| Nigral predominant-type LBs | 0.75 (0.30, 1.86), p=0.544 | 0.79 (0.32, 1.96), p=0.625 |
| Limbic-type LBs | 1.03 (0.71, 1.48), p=0.859 | 1.02 (0.70, 1.46), p=0.912 |
| Neocortical-type LBs | 1.73 (1.27, 2.36), p<0.001 | 1.70 (1.24, 2.31), p<0.001 |
| Neuritic plaques burden | 1.56 (1.37, 1.79), p<0.001 |  |
| Neurofibrillary tangles burden |  | 1.59 (1.38, 1.83), p<0.001 |
| APOE ε4 | 1.87 (1.44, 2.42), p<0.001 | 2.01 (1.55, 2.60), p<0.001 |

Model 1 was adjusted for age at death, sex, education, neuritic plaques burden, and APOE ε4.

Model 2 was adjusted for age at death, sex, education, neurofibrillary tangles burden, and APOE ε4.

**Table 2: Association of neocortical-type Lewy bodies with LATE-NC, stratified by age (above and below 90 years).**

| Predictor | Age-at-death | |
| --- | --- | --- |
|  | Below 90 years  OR (95% CI), p-value | 90 years and above  OR (95% CI), p-value |
| Nigral predominant-type LBs | 0.22 (0.02, 1.87), p=0.167 | 1.34 (0.45, 4.01), p=0.593 |
| Limbic-type LBs | 0.52 (0.27, 1.01), p=0.063 | 1.44 (0.91, 2.26), p=0.113 |
| Neocortical-type LBs | 2.26 (1.43, 3.55), p<0.001 | 1.34 (0.90, 2.00), p=0.147 |

A logistic regression model was adjusted for sex, education, and AD pathology.

**Table 3: Association of neocortical-type Lewy bodies with LATE-NC, stratified by sex.**

| Predictor | Sex | |
| --- | --- | --- |
|  | Female  OR (95% CI), p-value | Male  OR (95% CI), p-value |
| Nigral predominant-type LBs | 0.97 (0.32, 2.96), p=0.967 | 0.48 (0.10, 2.31), p=0.363 |
| Limbic-type LBs | 1.12 (0.73, 1.70), p=0.592 | 0.79 (0.39, 1.60), p=0.527 |
| Neocortical-type LBs | 2.20 (1.52, 3.19), p<0.001 | 0.99 (0.57, 1.71), p=0.977 |

A logistic regression model was adjusted for age, education, and AD pathology.

**Table 4: Association of neocortical-type Lewy bodies with LATE-NC, stratified by people with and without pathologic diagnosis of AD.**

| Predictor | Pathologic diagnosis of AD | |
| --- | --- | --- |
|  | With AD  OR (95% CI), p-value | Without AD  OR (95% CI), p-value |
| Nigral predominant-type LBs | 0.85 (0.28, 2.56), p=0.778 | 0.76 (0.16, 3.64), p=0.736 |
| Limbic-type LBs | 1.16 (0.77, 1.75), p=0.471 | 0.88 (0.41, 1.91), p=0.763 |
| Neocortical-type LBs | 1.62 (1.15, 2.29), p=0.005 | 2.12 (1.13, 3.98), p=0.019 |

A logistic regression model was adjusted for age, sex, education, and APOE ε4.
